# Role of Berberine in the Treatment of Methicillin-Resistant *Staphylococcus aureus* Infections

**DOI:** 10.1038/srep24748

**Published:** 2016-04-22

**Authors:** Ming Chu, Ming-bo Zhang, Yan-chen Liu, Jia-rui Kang, Zheng-yun Chu, Kai-lin Yin, Ling-yu Ding, Ran Ding, Rong-xin Xiao, Yi-nan Yin, Xiao-yan Liu, Yue-dan Wang

**Affiliations:** 1 Department of Immunology, School of Basic Medical Sciences, Peking University, Beijing, 100191, China; 2 Key Laboratory of Medical Immunology, Ministry of Health, Beijing, 100191, China; 3 Pharmacy Departments, Liao Ning University of Traditional Chinese Medicine, Liao Ning, 116600, China; 4 Department of Pathology, the First Affiliated Hospital of General Hospital of Chinese People’s Liberation Army, Beijing, 100048, China

## Abstract

Berberine is an isoquinoline alkaloid widely used in the treatment of microbial infections. Recent studies have shown that berberine can enhance the inhibitory efficacy of antibiotics against clinical multi-drug resistant isolates of methicillin-resistant *Staphylococcus aureus* (MRSA). However, the underlying mechanisms are poorly understood. Here, we demonstrated that sub-minimum inhibitory concentrations (MICs) of berberine exhibited no bactericidal activity against MRSA, but affected MRSA biofilm development in a dose dependent manner within the concentration ranging from 1 to 64 μg/mL. Further study indicated that berberine inhibited MRSA amyloid fibrils formation, which consist of phenol-soluble modulins (PSMs). Molecular dynamics simulation revealed that berberine could bind with the phenyl ring of Phe19 in PSMα2 through hydrophobic interaction. Collectively, berberine can inhibit MRSA biofilm formation *via* affecting PSMs’ aggregation into amyloid fibrils, and thereby enhance bactericidal activity of antibiotics. These findings will provide new insights into the multiple pharmacological properties of berberine in the treatment of microbial-generated amyloid involved diseases.

Berberine is an isoquinoline alkaloid presented in various plants^1^, such as *Rhizoma coptidis*^2^, which has been widely used to treat bacterial diarrhea and gastroenteritis for a long history^3^,^4^. Recently, berberine has been demonstrated to be a strong synergist for antibiotics^5^,^6^. Synergistic interactions between berberine and commonly used antimicrobial agents exhibit therapeutic benefits against a broad spectrum of pathogenic microorganisms, including methicillin-resistant *Staphylococcus aureus* (MRSA)^7^,^8^,^9^,^10^. Many reports have shown that combined use of berberine improved the bactericidal activity of antibiotics against MRSA, lower the MICs of antibiotics, and notably decreased adhesion and intracellular invasion of MRSA^8^,^9^,^10^.

MRSA is one of the most commonly recognized antibiotic-resistant bacteria, which is associated with high morbidity and mortality. The spread of MRSA is of great concern in the treatment of nosocomial infections, since it has quickly acquired resistance to most antibiotics^11^. Recent studies revealed that the antibiotic resistance capabilities of MRSA are associated with biofilm formation, which causes treatment failure and recurrent infections^12^,^13^,^14^. Biofilm acts as a barrier to antimicrobial agents and protect the colonies from any fluctuations of the environment. Microbial biofilm is a structured community of microbial cells enclosed in a self-produced polymeric matrix and adherent to an inert or living surface^15^. An extracellular amyloid fibril has been discovered in all MRSA biofilm matrix, which are composed of small peptides called phenol-soluble modulins (PSMs). PSMs have recently emerged as a novel toxin family contributing to MRSA biofilm development and the dissemination of biofilm-associated infections^16^,^17^,^18^. Notably, ordered aggregation of PSM peptides into amyloid fibrils can abrogate the biofilm disassembly activity ascribed to monomeric PSM peptides^16^. In this study, we investigated the capacity of berberine to inhibit MRSA biofilm formation, and gained insights into the underlying molecular mechanisms.

## Results

### Bactericidal activity of berberine

**Figure 1:**
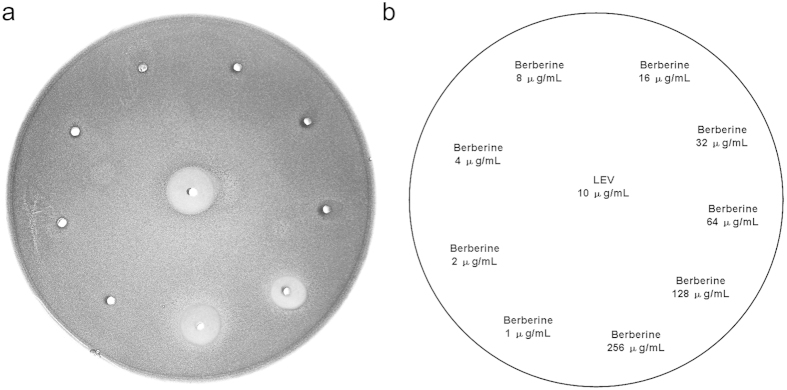
Bacteriostatic activity of berberine against MRSA-ATCC 33591. MRSA was inoculated into BHI broth and incubated with various concentrations of berberine. LEV (16 μg/mL) was used as positive control. Inhibition zones were photographed after 48 h incubation at 37 °C. (**a**) shows the plain agar plates, (**b**) describes the images for (**a**).

The susceptibility testing showed that the MIC value of berberine against the tested MRSA-ATCC 33591 strain was 128 μg/mL. It is noted that berberine had no bactericidal activity within the dose range from 1 to 64 μg/mL, but showed a powerful bacteriostatic effect against MRSA on the plain agar plate at 128 and 256 μg/mL (Fig. 1). The mean diameter of the inhibition zone for 128 and 256 μg/mL berberine was 8 and 12 mm, respectively.

### Inhibitory effect of berberine on MRSA biofilm formation

**Figure 2:**
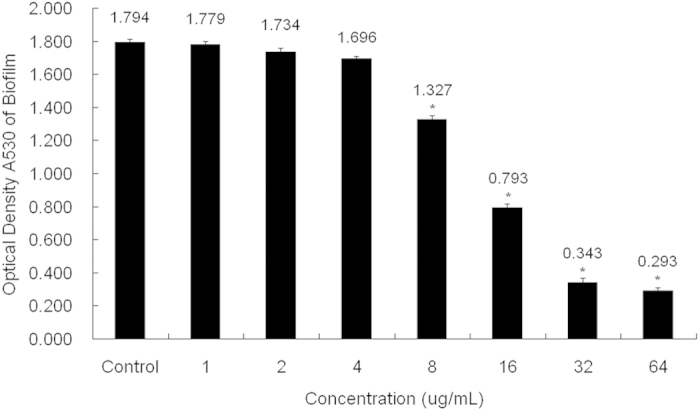
Inhibitory effects of berberine on MRSA biofilm formation. MRSA was inoculated into BHI broth and incubated with various concentrations of berberine. The biofilms that formed on the dish surface were measured by staining with 0.1% safranin. The bound safranin was released from the stained cells with 30% acetic acid. Data are represented as mean ± standard deviation. *Significance was determined at P < 0.05 when compared with the control.

Biofilms play an intrinsic role in protecting MRSA from any potential antimicrobial agents. Biofilm formation was studied using safranin staining, and the absorbance was measured at 530 nm. The negative control showed an OD of 1.794, whereas experimental groups treated with berberine at 1, 2, 4, 8, 16, 32, and 64 μg/mL showed ODs of 1.779, 1.734, 1.696, 1.327, 0.793, 0.343, and 0.293 respectively (Fig. 2).

**Figure 3:**
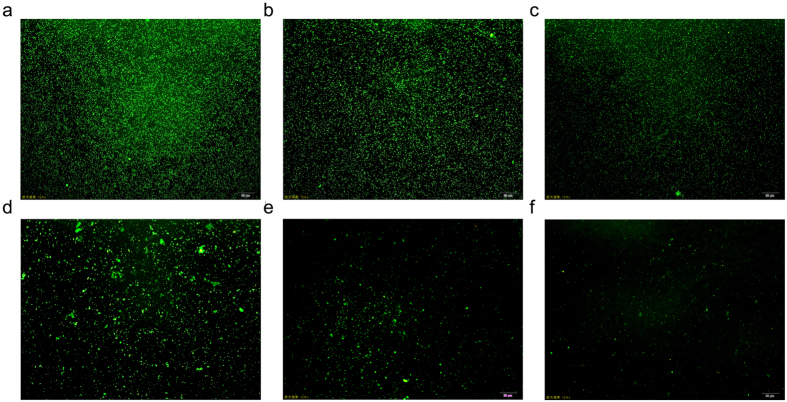
CLSM analysis of MRSA biofilms. MRSA was incubated with different concentrations of berberine (**a**) control; (**b**) 4 μg/mL; (**c**) 8 μg/mL; (**d**) 16 μg/mL; (**e**) 32 μg/mL; (**f**) 64 μg/mL; scale bar = 50 μm. MRSA biofilms were stained with acridine orange, and observed with CLSM at a magnification of 200×.

Acridine orange is a strong fluorescent biofilm biomass indicator that can stain all microbial colonies in a biofilm, alive or dead. Therefore, we observed the biofilm using a confocal laser scanning microscope (CLSM). CLSM images showed that in the culture without berberine, MRSA produced numerous microbial colonies covering the entire surface of the coverslips. In the culture with 8 μg/mL berberine, the number of colonies was substantially decreased and presented a discrete distribution. A further decrease in colonies occurred in the culture with 16 μg/mL berberine. When the concentration of berberine was over 32 μg/mL, very few colonies were present on the surface coverage of coverslips (Fig. 3).

**Figure 4:**
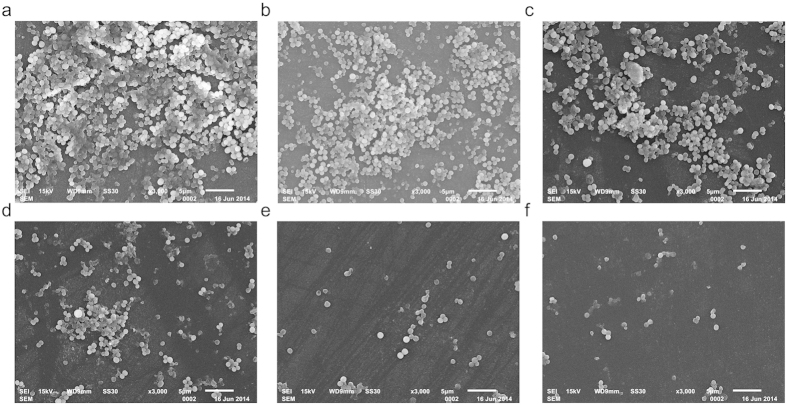
SEM analysis of MRSA biofilms. SEM images of biofilms formed by MRSA incubated with various concentrations of berberine (**a**) control; (**b**) 4 μg/mL; (**c**) 8 μg/mL; (**d**) 16 μg/mL; (**e**) 32 μg/mL; (**f**) 64 μg/mL; scale bar = 5 μm.

The morphology of MRSA biofilm on the surface of coverslips was observed using SEM. Under a 5,000× magnification, biofilm was shown to be composed of many multilayered MRSA colonies. SEM analysis results were consistent with those of acridine orange staining observations. As the concentration of berberine increased, we observed complete biofilm eradication in a dose dependent manner, and an obvious decrease in the density of MRSA (Fig. 4).

### Effect of berberine on microbial amyloid fibril formation

**Figure 5:**
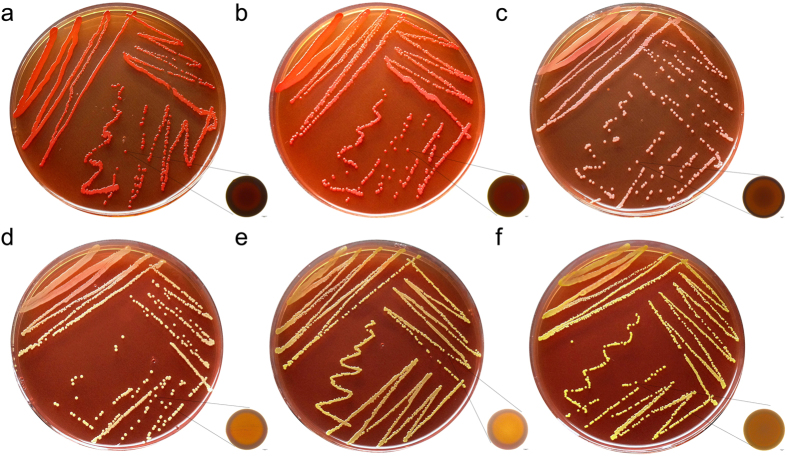
Congo red staining of MRSA amyloid fibril. MRSA was grown on Congo red medium and incubated with various concentrations of berberine (**a**) control; (**b**) 4 μg/mL; (**c**) 8 μg/mL; (**d**) 16 μg/mL; (**e**) 32 μg/mL; (**f**) 64 μg/mL; scale bar = 100 μm. Amyloid-fibril-producing strains show distinctive red colonies.

Congo red is currently used as a sensitive diagnostic tool to visualize microbial-generated amyloids. When grown in berberine-free medium, the MRSA strain showed a classical red, dry and rough phenotype on Congo red-supplemented agar, indicating normal amyloid fibril formation. In the culture with 8 μg/mL berberine, smooth and pink colonies were observed for MRSA producing very few amyloid fibrils. A further decrease of amyloid fibrils was observed in the MRSA colonies which were cultured with 16 μg/mL berberine. Non-amyloid-fibril-producing colonies were observed for MRSA when the concentration of berberine was over 32 μg/mL (Fig. 5).

**Figure 6:**
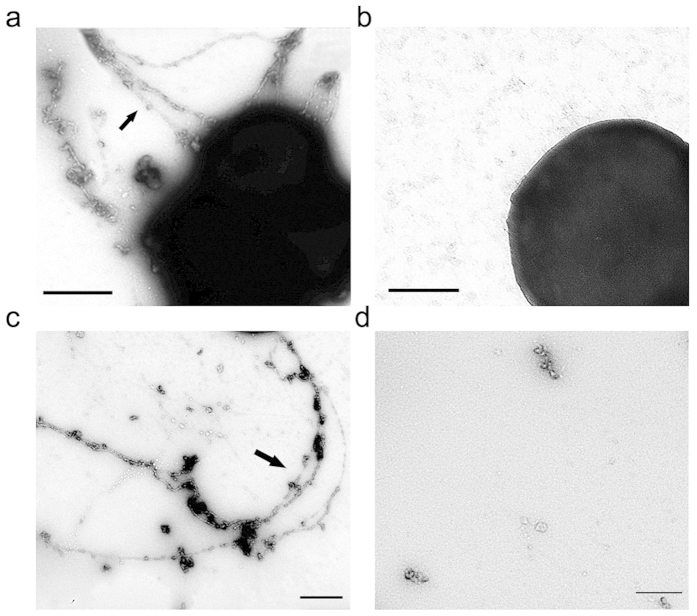
Inhibitory effect of berberine on MRSA amyloid fibril. TEM micrographs of cells from MRSA biofilms in the culture with berberine (**a**) control; (**b**) 32 μg/mL. (**c**) 24 hours after mixing 100 μg/mL each of the seven PSM peptides (α1-4, β1-2, and δ-toxin), fibril structures were readily observed by TEM. (**d**) Few amyloid fibril was observed when PSM peptides were cultured with 32 μg/mL berberine. Bars indicate 500 nm.

TEM imaging of cells revealed the presence of extracellular amyloid fibrils in MRSA biofilms. MRSA has been shown to produce large, extracellular structures (Fig. 6a). When in the culture with 32 μg/mL of berberine, few amyloid fibrils presented in the MRSA biofilms (Fig. 6b). How does berberine affect amyloid fibril formation? It is known that PSMs can aggregate into the amyloid fibrils in MRSA and modulate their ability to influence the biofilm formation^16^. Therefore, we wondered if berberine can affect PSMs polymerization. Synthetic PSM peptides were allowed to polymerize overnight (Fig. 6c). It is noted that berberine can prevent PSM peptides from aggregating into amyloid fibrils at the concentration of 32 μg/mL (Fig. 6d).

### Molecular dynamics simulation on the interaction between berberines and a PSM monomer

**Figure 7:**
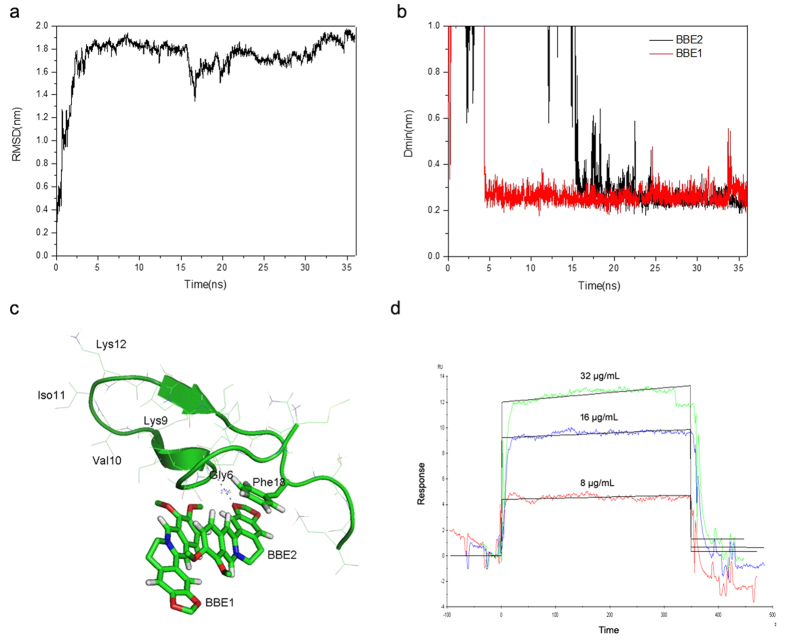
Interaction between berberine and PSMα2. (**a**) shows the backbone RMSD of PSMα2 during a 36 ns simulation. (**b**) The minimum distances between berberines (BBE1 and BBE2) and PSMα2 throughout the simulation. (**c**) The final structure of the simulation was visualized using the PyMOL Molecular Graphics System. (**d**) The association and dissociation of berberine with synthetic PSMα2 peptide was analyzed using SPR sensor chip.

To investigate the interaction between berberine and PSMs, we performed a molecular dynamics (MD) simulation using one monomer of PSMs exemplified by PSMα2, two berberines and 20210 water molecules. During a 36 ns simulation, the root-mean-square deviation (RMSD) of PSMα2 backbone rose up to 1.8 nm at 5 ns, and fluctuated around 1.8 nm for the rest of the simulation time, indicating that the PSMα2 has folded into a stable conformation (Fig. 7a). Meanwhile, the minimum distance (D_min_) between PSMα2 and berberine was measured throughout the simulation (Fig. 7b). Initially, the D_min_s for both of the two berberines were above 1.0 nm, implying that the two berberines were in a state of random diffusion. Then, the D_min_ for one berberine (BBE1) drops to 0.25 nm at 5 ns, and the other berberine (BBE2) drops to 0.25 nm at 15 ns. Of note, the D_min_ for both berberines kept vibrating around the value until the end of simulation, which indicated that both of the two berberines can bind to PSMα2 and formed a stable complex.

To gain insights into the detailed binding mode between the two berberines and PSMα2, we visualized the final structure of the simulation using the PyMOL Molecular Graphics System. As shown in Fig. 7c, the PSMα2 folded from an extended conformation into a U-shaped structure, with a β-turn in the middle of the peptide, involving residues of Lys9, Val10, Iso11 and Lys12. These four residues adjacent to the β-turn formed a short anti-parallel β-strand. It is noted that both of the two berberines can stack onto the phenyl ring of Phe19 in the PSMα2. In addition, a weak hydrogen bond formed between one of the berberines and the NH group from the backbone of Gly6 in the PSMα2. The binding activity of berberine to PSMα2 was further confirmed using a Surface Plasmon Resonance (SPR) biosensor chip (Fig. 7d). Collectively, berberine might prevent PSMs aggregation *via* disruption of the intermolecular attractions among PSM monomers.

## Discussion

Berberine is a natural isoquinoline alkaloid drawing increased attention for its multiple therapeutic effects on cancer, diabetes, hyperlipidemia, cardiovascular diseases, and central nervous system (CNS) disorders^19^,^20^,^21^,^22^,^23^. In traditional medicine, berberine has been widely used to treat bacterial diarrhea and gastroenteritis for a long history. Recently, berberine was demonstrated as a strong synergist for many commonly used antibiotics against clinical multi-drug resistant isolates of MRSA^8^,^9^,^10^. It is noted that sub-MICs of berberine exhibited significant synergistic effects on four conventional antimicrobial agents, including ampicillin (AMP), azithromycin (AZM), cefazolin (CFZ), and LEV^9^. However, the underlying mechanisms are poorly understood. Our study revealed that the MICs of berberine against MRSA-ATCC 33591 strain was 128 μg/mL with no inhibitory effect within the concentration ranging from 1 to 64 μg/mL. Thus, there might be other possible mechanisms in the synergistic action of berberine besides bactericidal activities.

As known, the antibiotic resistance capabilities of MRSA are associated with biofilm formation. Recent studies have shown that berberine possesses anti-biofilm activity against a broad spectrum of pathogenic microorganisms, such as *S. epidermidis, C. albicans, Salmonella Typhimurium*, and *S. aureus*^24^,^25^,^26^,^27^,^28^. Thus, we further investigated the anti-biofilm activity of berberine against MRSA. Biofilm assay revealed that berberine can inhibit the MRSA biofilm formation significantly at the concentrations greater than 8 μg/mL. As the concentration of berberine increased, the number of microbial colonies in the biofilm decreased in a dose dependent manner. It is likely that sub-MICs of berberine possessed promising anti-MRSA activity *via* inhibition of biofilm formation.

The biofilm forming ability of MRSA was reported to be mediated by PSMs, which can be found in biofilms as fibrils with Congo red binding capacities similar to known amyloid proteins, such as amyloid-β peptide (Aβ) in Alzheimer’s disease. Soluble PSMs have a helical structure in solution, but transition to adopt a β-rich structure after aggregation^16^. Congo red staining for amyloidosis indicated that berberine affected PSMs’ aggregation into amyloid fibrils at the concentrations greater than 8 μg/mL. When in the culture with 32 μg/mL of berberine, few amyloid fibrils presented in the MRSA biofilms. This is presumably mediated by berberine *via* disruption of the intermolecular attractions among PSM monomers. Thus, we gained our insights into the interaction between berberine and PSMα2, which shares a conserved phenylalanine residue with other PSM monomers involved in the amyloid fibrils formation^16^. MD simulation showed that berberines can stack on the phenyl ring of Phe19 in PSMα2 through hydrophobic interaction. Since the π-π stacking between phenylalanine residues from two adjacent β strands is crucial for the aggregation of Aβ-like protein^29^,^30^, we believed that berberine can prevent PSMs aggregation through destabilizing the π-π stacking of phenylalanine residues.

It is noted that PSMs can modulate biofilm disassembly using amyloids aggregation as a control point for their activity^16^. Soluble PSMs disperse biofilms while polymerized PSM peptides enhanced biofilm formation^17^,^18^. This work presents evidence that berberine can inhibit MRSA biofilm formation by affecting self-assembling of PSMs into amyloid fibrils, and thus enhancing bactericidal activity of antibiotics. The production of microbial amyloids may be a shared feature of biofilm matrices from many different microbial communities, such as such as curli in *Escherichia coli*, TasA in *Bacillus subtilis*, and the Fap fimbriae in *Pseudomonas aeruginosa*^31^,^32^,^33^. Thus, we believe that our research will lead to new approaches in treating persistent biofilm associated infections.

Moreover, berberine has recently gained much attention for its multiple pharmacological properties mediated by targeting molecules, which are involved in a wide range of biological processes, molecular functions and signaling pathways^34^,^35^,^36^,^37^. In addition to microbial amyloids, berberine was reported to prevent Aβ aggregation and alter amyloid precursor protein processing^37^, which indicated that berberine may act as a promising agent to combat Alzheimer’s disease^38^. Therefore, we propose that further study in the interaction between berberine and microbial-generated amyloids will provide new insights into the potential therapeutic effects of berberine in the treatment of amyloid-involved diseases.

## Methods

### Strains and growth conditions

Methicillin-resistant *S. aureus* (MRSA-ATCC 33591) strain was used in this study, because it is capable of biofilm formation *in vitro*^39^. MRSA was maintained in tryptic soy broth medium (TSB, Baltimore, MD, USA) and frozen at −80 °C until use. The microorganism was subcultured onto brain-heart infusion agar plates (BHI, Detroit, MI, USA) and incubated at 37 °C for 24 h to generate the bacteria cells for the experiments.

### Antimicrobial agents

Berberine (Sigma-aldrich, St. Louis, MO, USA) was dissolved in deionized water and filtered through a 0.22 μm Millipore filter (Sartorius Co., NY, USA)^40^,^41^,^42^. Levofloxacin (LEV) was obtained from Yangzhijiang Pharmaceutical Co. (Taizhou, China).

### Susceptibility testing

The MICs of berberine were determined by a microtitre broth dilution method^7^. Growth inhibition assays were performed in sterile 96-well plates (Corning Co., NY, USA) in a final volume of 200 μL, consisting of 100 μL of microbial cultures (5 × 10^5^ CFU/mL) and 100 μL of serially diluted berberine (2, 4, 8, 16, 32, 64, 128, 256, and 512 μg/mL). Microplates were incubated at 37 °C for 24 h, and the microbial cell growth was assessed by measuring the optical density of cultures at 600 nm wavelength with a Multiskan MK3 microplate reader (Thermo Fisher Scientific, Waltham, MA, USA). All samples were prepared in triplicates. MICs were defined as the lowest berberine concentration that yielded no visible growth after 24 h.

### Agar diffusion test

Microbial suspension was inoculated into BHI broth and kept for 30 min to allow broth cultures to stabilize. Then, berberine (1, 2, 4, 8, 16, 32, 64, 128, and 256 μg/mL) were added into the agar plate. LEV (16 μg/mL) was used as positive control. Inhibition zones were photographed after 24 h incubation at 37 °C^6^.

### Biofilm assay

The biofilm assay was based on a method described previously^43^. Berberine was added to the BHI broth containing 1% glucose in 24-well plates (Corning Co., NY, USA). The cultures were then inoculated with a seed culture of MRSA (5 × 10^5^ CFU/mL). After cultivating for 24 h at 37 °C, the supernatant was completely removed, and the wells or wells containing glass coverslips were rinsed with distilled water. The amount of biofilm formed in the wells was measured by staining with 0.1% safranin. The bound safranin was released from the stained cells with 30% acetic acid and the absorbance of the solution was measured at 530 nm. The biofilm formed on the surface of the glass coverslips was stained with 0.01% acridine orange and observed with a confocal laser scanning microscope (Leica, Heidelberg, Germany).

### Scanning electron microscopy

The biofilm on glass coverslips was also determined by scanning electron microscopy (SEM)^43^. The biofilm formed on the glass coverslips was rinsed with distilled H_2_O and fixed with 2.5% glutaraldehyde in 0.1 M sodium cacodylate buffer (pH 7.2) at 4 °C for 24 h. After gradual dehydration with ethylalcohol (60%, 70%, 80%, 90%, 95%, and 100%), the sample was freeze dried. The specimens were then sputter coated with gold. For observation, a TM3030 SEM (Hitachi, Tokyo, Japan) was used.

### Congo red agar method

Freeman *et al.* had described a Congo red agar (CRA) method for detecting the production of amyloids by *S. aureus*^44^. The medium was composed of TSB 37 g/L, sucrose 50 g/L, and Congo red (AMERCO, Solon, OH, USA) 0.8 g/L. Congo red stain was prepared as a concentrated aqueous solution and autoclaved (121 °C for 15 min) separately from the other medium constituents, and was then added when the agar had cooled to 55 °C. Plates were inoculated and incubated aerobically for 24 h at 37 °C. Positive result was indicated by red colonies with a dry crystalline consistency. Non-amyloid-fibril-producing strains were usually remained pink, though darkening at the center of the colonies was observed.

### Transmission electron microscopy

Transmission electron microscopy (TEM) was performed using a JEM-2100 transmission microscope (JOEL, Tokyo, Japan). Samples prepared for TEM imaging were spotted onto formvar-coated copper grids, incubated for 5 mins, washed with sterile distilled H_2_O, and negatively stained with 2% uranyl acetate for 60 seconds^9^.

### PSM polymerization experiments

PSM peptides (α1-4, β1-2, and δ-toxin) were synthesized and prepared as previously described^16^. All assays were performed with equal stoichiometric ratios of 0.1 mg/mL peptide. Synthetic PSM peptides were allowed to polymerize overnight and fibril formation was verified by TEM imaging.

### Molecular dynamics simulation

The MD simulation was performed using Gromacs 4.5 package^45^. Gromos96 (53A6) force field was used for the PSMα2^46^. The parameters for the berberine were generated with PRODRG web server^47^. Two berberines were put beside the PSMα2 with a distance over 1.5 nm. A cubic water box with the length of 9 nm in each side was used to solvate the berberines and PSMα2. Four Cl^−^ ions were added to keep the system neutral in charge. The system was minimized for 1000 steps with the steep decent method, and simulated at 300 K under NPV condition for 100 ps to adapt its density. Then, 36 ns product simulation was conducted under NPT condition. Weak coupling method was used with coupling constants of 0.1 ps and 0.5 ps respectively to maintain the systems at 300 K and 1 Bar. The bonds length involved in the PSMα2 and berberines were constrained with LINCS, while those in waters were constrained with SETTLE algorithm. Particle Mesh Ewald (PME) method was used to calculate electrostatic interactions beyond the cut off at 10 nm. Van der Waals interactions were cut off at distance of 14 nm and updated every 10 steps. Snapshots of trajectories were saved every 10 ps for analysis.

### Surface Plasmon Resonance biosensor analysis

Biacore T-100 machine was used for analysis. Synthetic PSMα2 peptides (fMGIIAGIIKFIKGLIEKFTGK) were immobilized on a sensor chip^48^. Berberine was initially dissolved in 0.1 M sodium acetate buffer of pH 5.0, and further diluted using the running buffer, ranging from 8 μg/mL to 32 μg/mL. The flow rate was 30 μL/min. The kinetic parameters were computed using Biacore T-100 evaluation software.

### Statistical analysis

The statistical analyses were conducted using Student’s t-test with SPSS 13.0 software^49^. The data were expressed as the means ± standard deviation. Values of *p* < 0.05 were considered statistically significant.

## Additional Information

**How to cite this article**: Chu, M. *et al.* Role of Berberine in the Treatment of Methicillin-Resistant *Staphylococcus aureus* Infections. *Sci. Rep.* **6**, 24748; doi: 10.1038/srep24748 (2016).
